# Acute Myeloid Leukemia with Concomitant BCR-ABL and NPM1 Mutations

**DOI:** 10.1155/2019/6707506

**Published:** 2019-04-11

**Authors:** Benedetta Mariotti, Federico Meconi, Raffaele Palmieri, Eleonora De Bellis, Serena Lavorgna, Tiziana Ottone, Vincenza Martini, Francesco Lo-Coco, Laura Cicconi

**Affiliations:** ^1^Department of Biomedicine and Prevention, University Tor Vergata, Rome, Italy; ^2^UOC Ematologia, Ospedale “Fabrizio Spaziani”, Frosinone, Italy

## Abstract

We present a case report of a patient with acute myeloid leukemia (AML) characterized by the simultaneous presence of nucleophosmin 1 (NPM1) mutation and the breakpoint cluster region-Abelson (BCR-ABL) fusion oncogene. Our findings emphasize the importance of routinely including BCR-ABL in the diagnostic workup of AML in order to offer to the patients the most appropriate risk category and treatment options.

## 1. Introduction

Mutations in the *NPM1* gene are the most frequent genetic abnormalities occurring in AML and are highly specific for *de novo* AML [1]. The BCR-ABL fusion gene is the genetic hallmark of chronic myeloid leukemia (CML) but can also be found in approximately 30% of acute lymphoblastic leukemia (ALL) and rarely in AML (0.3–2% of newly diagnosed cases). In the updated WHO classification published in 2016, AML with BCR-ABL has been introduced as a provisional new entity [2, 3]. In BCR-ABL-positive AML, molecular and cytogenetic profile may help to distinguish *de novo* AML cases from CML-blastic phase (CML-BP).

To the best of our knowledge, the co-occurrence of *BCR-ABL* fusion gene and *NPM1* mutations in *de novo* AML has been reported in only few cases [4]. Herein, we report a 74-year-old patient diagnosed with AML harboring a complex three-way translocation t(9;22;12)(q34;q13;q11) encoding for two isoforms of BCR-ABL transcript (b3a2;b2a2) and a concomitant type A mutation in the *NPM1* gene.

## 2. Case Report

**Figure 1:**
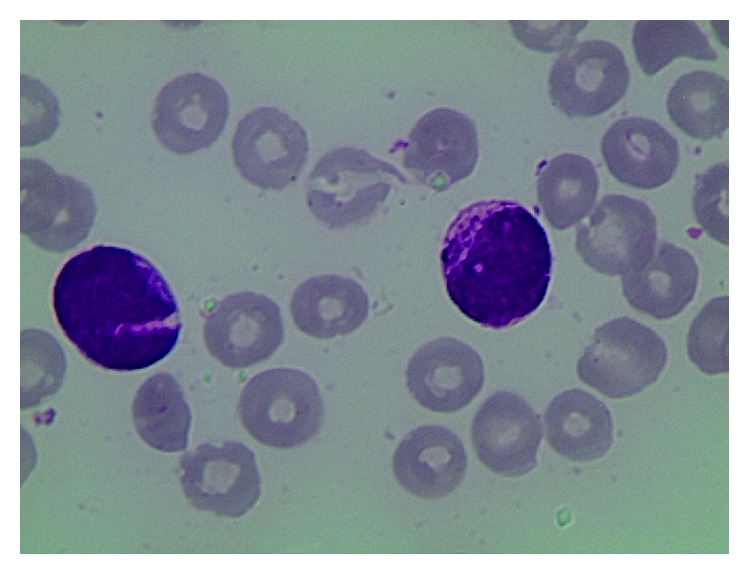


**Figure 2:**
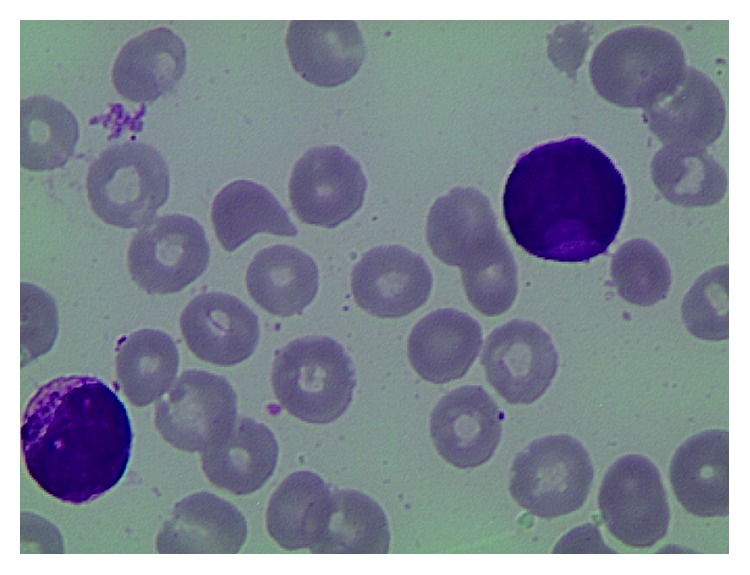


A 74-year-old male, with a history of type II diabetes and previous ischemic heart disease, was admitted on September 2014 to the emergency room of the hospital complaining severe asthenia and nasal bleeding. There was no previous history of hematological disorders. Blood cell count disclosed Hb 6.4 gr/dL (12.0–16.0 g/dL), Plts 35 × 10^9^/L (150–450 × 10^9^/L), a WBC of 62 × 10^9^ (4.30–10.80 × 10^9^/L), basophils <2% (0–1.5%), and with 50% of blasts. The coagulation profile showed INR 1.5 (0.8–1.2), fibrinogen 69 mg/dL (200–400 mg/dL), ATIII 77% (75–128%), and D-dimer 10757 ng/mL (0–500 ng/mL), suggesting a disseminated intravascular coagulopathy (DIC). Bone marrow aspirate showed infiltration by 89% of hypergranular leukemic blasts (Figures 1 and 2).

Immunophenotyping of the leukemic population showed positivity for CD45, CD33, CD117, and MPO and negativity for CD34, HLA-DR, CD13, and CD56, compatible with a diagnosis of AML. Clinical examination showed mild splenomegaly (14 cm) and multiple thick and erythematous skin lesions localized on the back. A biopsy of one such lesion followed by histologic examination was consistent with extramedullary localization of AML.

**Figure 3:**
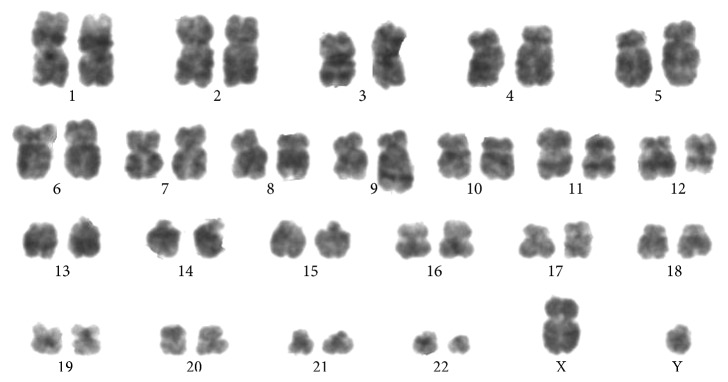
G-banding karyotype from a bone marrow blood metaphase.

**Figure 4:**
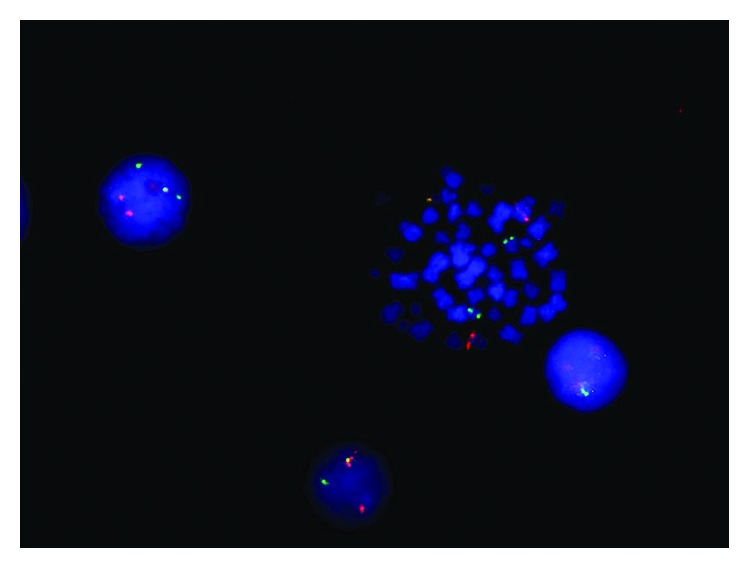
FISH BCR/ABL dual color dual fusion probe.

Conventional karyotyping (Figure 3) and FISH (Figure 4) showed the presence of a three-way translocation t(9;12;22)(q34;q13;q11) on 15/15 metaphases.

The p210 BCR-ABL fusion transcript was detected by standard RT-PCR, which allowed to identify both b3a2 and b2a2 transcript isoforms [5]. Nowadays, no data are available regarding prognostic value of these transcripts in AML; however, studies regarding CML have shown that in some cases transcript, b2a2 has slower molecular and inferior response rates to TKI and a poorer long‐term outcome [6]. In addition, molecular screening allowed to detect the presence of a type A mutation in the *NPM1* gene [7] and the absence of BCR/ABL p190, RUNX1/RUNXT1, CBFbeta/MYH11, DEK/CAN, FLT3-ITD, and PML/RARalpha rearrangements. Quantitative RQ-PCR showed IS 36% of BCR-ABL, with a copy number of BCR-ABL p210 of 7,152 · 10^4^/ABL [8] and NPM1 copies of 101,160 · 10^4^/ABL [7].

The CT scan showed hepatic lesions suggestive for extramedullary involvement and disseminated venous thrombosis localized in the sovrahepatic veins and the right frontal sinus. Lumbar puncture showed no central nervous system involvement. The patient was started on initial cytoreductive treatment with hydroxyurea for ten days and was subsequently treated with second-generation tyrosine kinase inhibitor (TKI) dasatinib 140 mg PO daily. Dasatinib was preferred over imatinib, a first generation TKI, due to high risk of the central nervous system involvement, extramedullary localization [9, 10] and, as proven for CML, it gives deeper and longer molecular responses [11].

In December 2014, due to trilineage pancytopenia, research for point mutations in BCR/ABL fusion gene was detected and showed, unfortunately after patient died, T315I mutation.

In January 2015, three months after the start of dasatinib, he was hospitalized for neutropenic fever and was diagnosed with pneumonia. He died of sepsis few days later.

## 3. Discussion

To the best of our knowledge, only few cases of AML with concomitant *BCR-ABL* rearrangement and *NPM1* mutation have been reported so far [4]. In clinical practice, the distinction between *de novo* BCR-ABL +ve AML and CML-BP is still challenging [12, 13]. In the present case, patient clinical features (no history of previous hematologic disorders, lack of basophilia, mild splenomegaly, and a lower bone marrow myeloid/erythroid ratio [14]) and molecular abnormalities (concomitant presence of *NPM1* mutation) were more suggestive of a *de novo* AML rather than of CML-BP. In previous reports of simultaneous *NPM1* mutation and *BCR-ABL* rearrangement, the p210 and p190 transcripts were detected in 2 and 4 cases, respectively. However, the concomitant presence of b3a2 and b2a2 BCR-ABL p210 isoforms combined to simultaneous NPM1 mutation, as observed in the present report, has been reported to date in only one case [12]. An additional peculiarity detected in leukemic cells of our patient is the presence of a complex three-way translocation involving chromosomes 9, 22, and 12 a karyotypic aberration previously unreported in either AML or CML.

*BCR-ABL* seems to cooperate with several AML-specific aberrations, including *CBFB-MYH11* and *NPM1*, although the precise molecular interaction among the altered proteins remains poorly understood [15].

In our case, RQ-PCR analysis of NPM1 and BCR-ABL detected in both instances a high transcript copy number. Together with the elevated number of metaphases harboring the Philadelphia chromosome (15/15), these findings suggest that the two molecular aberrations were present within the same leukemic clone rather than occurring in two separate clones. However, in recently reported patients with Ph-positive AML, targeted treatment with TKIs was able to abrogate the BCR-ABL +ve clone but did not lead to complete hematologic response, indicating that the *BCR-ABL* lesion was present at the subclonal level [16]. This highlights the more complex clonal architecture of Ph-positive AMLs as compared to CML. In addition, BCR-ABL has been described only occasionally as a minor subclone arising during AML progression. Unfortunately, due to early death of our patients, we were not able to assess the cytogenetic/molecular response to TKI therapy.

At the moment, there is no standardized treatment for patients with Ph-positive AML although few case reports suggested a favorable response to TKIs [17]. In light of the new ELN classification [2], our findings further highlight the need of including BCR-ABL screening in routine diagnostic workup of AML. *BCR-ABL* and *NPM1* mutation are considered distinct entities in the WHO classification of AML with recurrent genetic abnormalities. According to the updated ELN criteria, the presence of *BCR-ABL* fusion gene classifies AML in the adverse risk category while *NPM1* mutation is generally regarded as a favorable prognostic in absence of the *FLT3*-ITD aberration. This case report highlights the possibility to concomitantly detect these two alterations in rare cases of AML and emphasizes the importance of including BCR-ABL screening in routine AML diagnostic panel in order to better assign the patient to the correct risk category and targeted treatment.
